# Intraoperative application of FFR pressure wire and FLOW800 imaging as predictors of postoperative cerebral perfusion abnormalities in Moyamoya disease

**DOI:** 10.3389/fsurg.2026.1792515

**Published:** 2026-04-01

**Authors:** Huan Li, Shubin Tan, Mohamed Helmy, Donglei Song, Bin Xu, Wei Wang

**Affiliations:** 1 Department of Neurosurgery, Donglei Brain Hospital, Shanghai, China; 2 Department of Neurosurgery, Huashan Hospital, Fudan University, Shanghai, China

**Keywords:** cerebral perfusion, FLOW800, fractional flow reserve, hemodynamic monitoring, Moyamoya disease, STA–MCA bypass

## Abstract

**Objective:**

To evaluate whether intraoperative fractional flow reserve (FFR) pressure wire measurements combined with FLOW800 imaging analysis effectively predict postoperative cerebral perfusion abnormalities following superficial temporal artery to middle cerebral artery (STA–MCA) bypass surgery in patients with Moyamoya disease (MMD).

**Methods:**

A retrospective analysis was conducted on 26 patients diagnosed with MMD who underwent STA–MCA bypass at our institution between November 2023 and January 2025. Intraoperative graft pressures were assessed using FFR pressure wires. Concurrently, FLOW800 imaging provided quantitative microcirculatory parameters, including delay time (DT), flow velocity, rise time (RT), and fluorescence intensity. Postoperative cerebral perfusion-related complications were documented. ROC analyses were reported with 95% confidence intervals to evaluate the predictive value of intraoperative parameters.

**Results:**

Postoperative cerebral perfusion abnormalities occurred in 9 out of 26 patients (34.6%). Among them, 3 patients (11.5%) had diffusion-weighted MRI (DWI)-confirmed acute ischemic lesions (major complications), whereas the remaining 6 patients experienced transient neurological symptoms that completely resolved within 2 weeks to 1 month without radiographic infarction. A higher pressure gradient across the bypass graft (ΔP) and prolonged rise time (RT) in the proximal recipient artery significantly correlated with postoperative perfusion abnormalities (*p* < 0.05). A ΔP cutoff >32 mmHg showed a sensitivity of 77.8% and a specificity of 64.7%. The combined predictive capability of ΔP and RT yielded an area under the receiver operating characteristic (ROC) curve (AUC) of 0.82 (95% CI, 0.61–0.99), surpassing the predictive value of either parameter alone (AUC 0.79 for ΔP and 0.79 for RT).

**Conclusions:**

Intraoperative monitoring with FFR pressure wire combined with FLOW800 imaging may help identify MMD patients at increased risk of early postoperative cerebral perfusion abnormalities after STA–MCA bypass. The integration of ΔP and RT appears to improve predictive accuracy and may support perioperative risk stratification, although larger prospective studies are required before routine decision-making can be recommended.

## Introduction

Moyamoya disease (MMD) is a chronic, progressive cerebrovascular disorder characterized by bilateral stenosis or occlusion of the supraclinoid internal carotid arteries and their proximal branches, leading to severe ischemic and hemorrhagic strokes (1, 2). Superficial temporal artery to middle cerebral artery (STA–MCA) bypass surgery has emerged as a well-established treatment modality aimed at restoring adequate cerebral perfusion. Despite its recognized efficacy, STA–MCA bypass surgery continues to be associated with postoperative cerebral perfusion complications, notably cerebral hyperperfusion syndrome (HPS) and focal hypoperfusion resulting in ischemia, which substantially affect patient outcomes and increase morbidity (3, 4).

Emerging evidence highlights that intraoperative hemodynamic alterations significantly correlate with postoperative cerebral perfusion outcomes (5). A rapid postoperative increase in cerebral blood flow following bypass surgery can surpass the brain's autoregulatory capacity, potentially leading to cerebral edema, hemorrhage, or ischemic injury (6). While conventional postoperative neuroimaging techniques, such as magnetic resonance imaging (MRI) or computed tomography (CT), effectively identify cerebral perfusion abnormalities *post hoc*, they do not facilitate real-time intraoperative monitoring and thus limit immediate preventive intervention.

Fractional flow reserve (FFR) pressure wire technology, traditionally employed in coronary interventions to measure intravascular pressure gradients with high accuracy, has recently been adapted to cerebrovascular procedures for precise intracranial hemodynamic assessments (7). Similarly, FLOW800 imaging, a novel intraoperative optical imaging technique employing indocyanine green fluorescence, provides real-time visualization and quantitative measurement of cerebral microvascular perfusion parameters (8).

Nevertheless, the integrated intraoperative application of FFR pressure wire measurements and FLOW800 imaging for STA–MCA bypass procedures in MMD remains underexplored. Limited data exist regarding the combined efficacy, predictive capability, and safety of these modalities. Therefore, this study aimed to comprehensively evaluate the combined predictive performance of intraoperative FFR pressure wire and FLOW800 imaging analyses for postoperative cerebral perfusion abnormalities. Such findings may provide critical evidence supporting refined intraoperative monitoring protocols and proactive surgical decision-making, ultimately improving patient safety and surgical outcomes in MMD.

## Materials and methods

### Patient selection

This retrospective study reviewed clinical data from 26 patients diagnosed with Moyamoya disease (MMD), who underwent superficial temporal artery to middle cerebral artery (STA–MCA) bypass surgery, from November 2023 to January 2025. Eligible patients were between 12 and 65 years of age and had a confirmed diagnosis of MMD based on digital subtraction angiography (DSA). All patients had clear surgical indications for revascularization and provided informed consent for intraoperative hemodynamic monitoring. Additional eligibility criteria included the presence of suitable recipient vessels for bypass and at least two branches of the superficial temporal artery (STA) accessible beneath the scalp flap.

Patients were excluded if they had Moyamoya syndrome secondary to other etiologies, underwent only indirect revascularization procedures (such as encephalo-myo-synangiosis), or had only one STA branch available for grafting.

### Ethics approval

This retrospective study was reviewed and approved by the Ethics Committee of Shanghai Donglei Brain Hospital (approval no. YW-LL-2023-001). The requirement for written informed consent was waived due to the retrospective nature of the study, in accordance with local legislation and institutional requirements.

### Surgical procedure and hemodynamic monitoring

**Figure 1:**
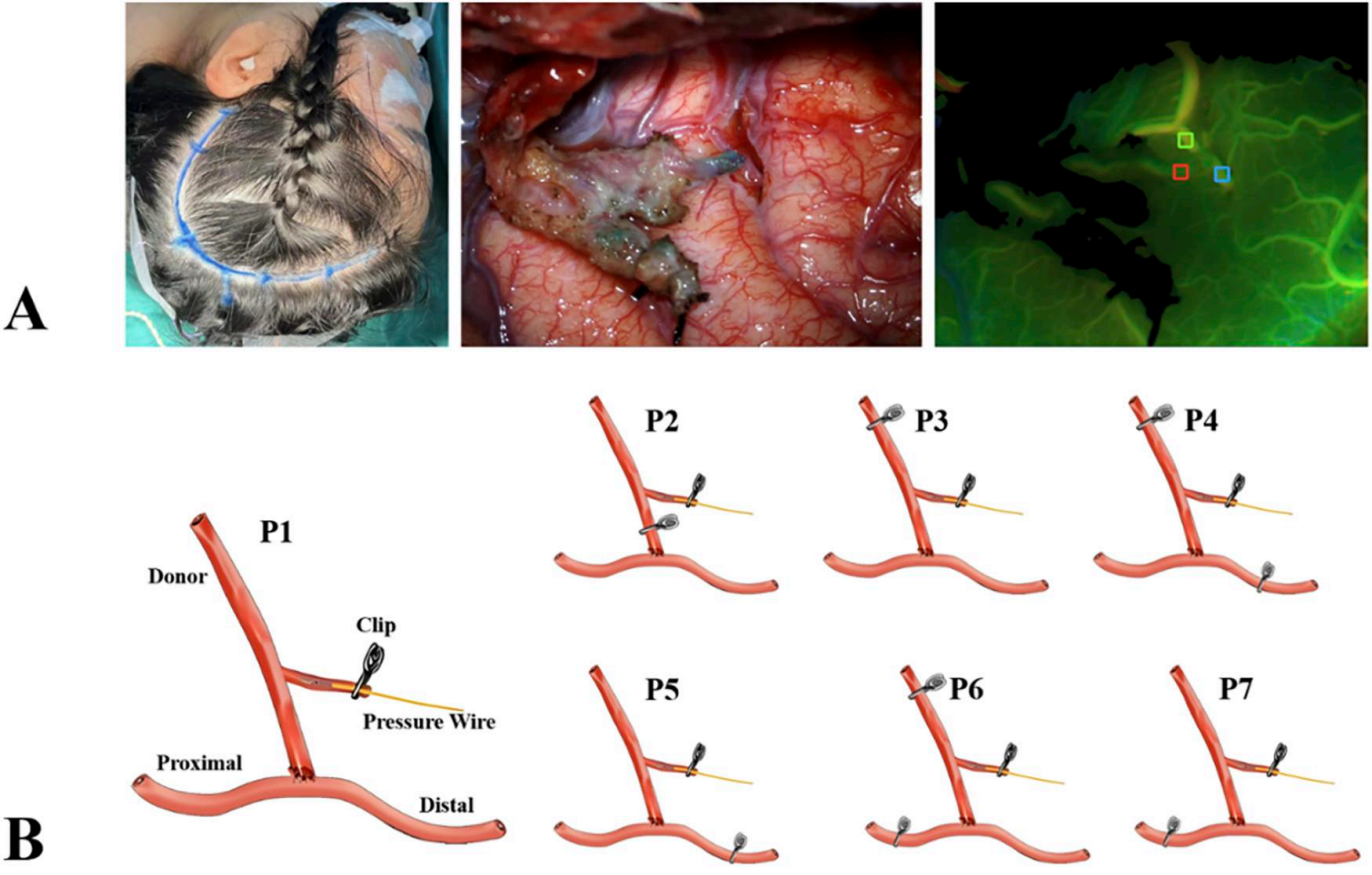
Study workflow and intraoperative measurement protocol. **(A)** Schematic illustration of the STA–MCA bypass procedure and pressure-wire measurement points, and FLOW800 imaging workflow for quantitative microcirculatory analysis. **(B)** Intraoperative placement of the FFR pressure wire for donor and recipient artery pressure assessment.

The study workflow, pressure-wire measurement points, and FLOW800 assessment are illustrated in Figure 1. Intraoperative hemodynamic measurements followed a standardized protocol developed by Helmy et al. (7) for extracranial-intracranial revascularization in MMD. A fractional flow reserve (FFR) pressure wire (Abbott) was inserted into the selected STA branch to measure pressure points (P1–P7) along with mean arterial pressure (MAP). Measurements were obtained under standardized general anesthesia with continuous hemodynamic monitoring and, whenever possible, during relatively stable systemic conditions. Post-measurement, the STA branch was ligated and electrocoagulated.

Simultaneous microcirculatory imaging was performed using the Zeiss FLOW800 system. Four microcirculatory parameters—delay time (DT), flow velocity, rise time (RT), and fluorescence intensity—were quantitatively measured in three defined regions of interest (ROIs) (9). Among these parameters, RT was of particular interest because it reflects the speed of fluorescence inflow and microvascular filling within the region of interest and may therefore be more sensitive to delayed microcirculatory adaptation after bypass. Postoperative blood pressure was maintained close to the preoperative baseline according to the institutional perioperative management protocol, and diffusion-weighted MRI (DWI) was used to evaluate ischemia if clinically suspected.

### Outcome definition

Postoperative cerebral perfusion abnormalities were defined as new neurological symptoms attributable to the operated hemisphere, with or without corresponding imaging changes. Given the limited sample size and heterogeneous mechanisms (including both hyperperfusion and hypoperfusion phenomena), these entities were not analyzed separately and were collectively termed “perfusion abnormalities”. Cases with DWI-confirmed acute ischemic lesions were additionally considered major complications.

### Statistical analysis

Statistical analyses were conducted using SPSS v29.0.1.0 (IBM Corp.). Continuous variables were presented as mean ± standard deviation (SD). Group comparisons utilized independent-sample *t*-tests, one-way ANOVA, median tests, or chi-square tests as appropriate. Receiver operating characteristic (ROC) curves and the area under the curve (AUC) evaluated the predictive accuracy of intraoperative parameters, and 95% confidence intervals were estimated by bootstrap resampling. Diagnostic performance of the proposed ΔP cutoff was summarized by sensitivity and specificity. A *p*-value <0.05 indicated statistical significance.

## Results

### Clinical characteristics

Intraoperative graft pressure data were collected from 26 patients (15 left-sided, 11 right-sided bypasses). No complications related to STA branch disconnection or wound healing were recorded. Clinical characteristics are summarized in Table 1.

**Table 1:** Clinical characteristics of Moyamoya disease patients.

Variable	Left side (*n* = 15)	Right side (*n* = 11)	*p*-value
Sex (male/female)	8/7	5/6	0.81
Age (years)	55.32 ± 12.45	57.89 ± 11.78	0.27
Pressure			
P1	78.40 ± 9.98	79.45 ± 11.06	0.80
P2	83.73 ± 7.41	82.18 ± 10.00	0.67
P3	40.33 ± 15.40	51.73 ± 17.51	0.10
P4	40.20 ± 14.08	50.55 ± 18.99	0.14
P5	78.60 ± 8.28	79.09 ± 11.11	0.90
P6	40.53 ± 17.11	46.36 ± 18.98	0.43
P7	78.40 ± 9.80	79.00 ± 9.96	0.88
Mean arterial pressure	84.80 ± 8.10	87.18 ± 4.42	0.35
Pressure difference			
ΔP	38.07 ± 11.61	27.73 ± 11.88	0.04
ΔP (proximal)	38.40 ± 10.13	28.55 ± 13.25	0.05
ΔP (distal)	37.87 ± 10.74	32.64 ± 14.29	0.32
Flow direction (Ante/Retro)	7/8	8/3	0.19
Suzuki stage			0.21
I	1	0	
II	3	2	
III	7	7	
IV	3	2	
V	0	0	
VI	1	0	
Donor artery			
DT	1.39 ± 0.82	1.42 ± 1.07	0.93
Speed	134.27 ± 107.14	73.73 ± 37.36	0.06
RT	3.50 ± 2.75	2.29 ± 1.57	0.17
Intensity	44.47 ± 24.66	58.82 ± 43.92	0.34
Receptor artery (proximal)			
DT	1.40 ± 1.19	1.35 ± 1.21	0.92
Speed	131.73 ± 97.14	206.91 ± 146.18	0.16
RT	4.09 ± 2.89	4.24 ± 3.95	0.91
Intensity	59.07 ± 18.17	55.91 ± 17.57	0.66
Receptor artery (distal)			
DT	1.11 ± 0.69	1.59 ± 1.08	0.21
Speed	213.27 ± 161.52	177.82 ± 151.74	0.57
RT	3.75 ± 2.23	4.43 ± 4.12	0.63
Intensity	64.07 ± 17.68	50.64 ± 22.53	0.12

The direction of recipient vessel flow was determined based on P4 and P6 parameters; P4 > P6 was considered anterograde flow; otherwise, retrograde. The pressure gradient between the pre-bypass and post-bypass (ΔP), calculated as the difference between donor pressure (P1) and recipient artery pressure (P3), was significantly higher in left-sided bypasses (38.07 mmHg) compared to right-sided (27.73 mmHg, *p* = 0.04). No significant differences were identified for other clinical or FLOW800-derived parameters between sides.

### Factors associated with postoperative perfusion abnormalities

Postoperative cerebral perfusion abnormalities were observed in 9 of the 26 patients (34.6%), presenting as new neurological deficits attributable to the operated hemisphere (e.g., contralateral limb weakness and/or aphasia). Diffusion-weighted MRI (DWI) confirmed acute ischemic lesions in 3 of these cases, representing a major complication rate of 11.5%. The remaining 6 patients showed no radiographic evidence of infarction and experienced complete neurological recovery within 2 weeks to 1 month. The remaining 17 patients exhibited no postoperative perfusion-related symptoms or imaging abnormalities.

As shown in Table 2, the pressure gradient across the bypass (ΔP) was significantly elevated in the perfusion abnormality group (*p* = 0.03), while the recipient artery pressure (P3) was notably lower (*p* = 0.02). No significant between-group difference in intraoperative mean arterial pressure was observed (86.5 ± 6.3 vs. 84.6 ± 7.9 mmHg, *p* = 0.55), suggesting that the association between ΔP and postoperative perfusion abnormalities was unlikely to be explained solely by systemic blood pressure at the time of measurement. FLOW800 analysis (Table 3) further revealed that patients with perfusion abnormalities demonstrated significantly prolonged rise time (RT) in the proximal recipient artery (*p* = 0.02), suggesting impaired local microvascular flow.

**Table 2:** Intraoperative pressure characteristics by postoperative cerebral perfusion status (mmHg).

Parameter	No abnormality (*n* = 17)	Abnomality (*n* = 9)	*p*-value
P1	80.82 ± 9.63	75.11 ± 10.89	0.21
P2	84.53 ± 8.90	80.33 ± 7.18	0.21
P3	50.71 ± 15.65	34.67 ± 14.92	0.02
P4	48.88 ± 16.29	36.44 ± 15.41	0.07
P5	80.06 ± 9.61	76.44 ± 8.95	0.35
P6	47.65 ± 17.11	34.22 ± 16.48	0.07
P7	80.71 ± 8.73	74.78 ± 10.69	0.17
Mean arterial pressure	86.45 ± 6.27	84.59 ± 7.90	0.55
ΔP	30.12 ± 12.66	40.44 ± 9.90	0.03
ΔP (proximal)	31.18 ± 12.68	40.00 ± 9.86	0.06
ΔP (distal)	33.06 ± 12.93	40.56 ± 10.15	0.12

**Table 3:** Intraoperative ICG parameters by postoprative cerebral perfusion status.

Parameter	No abnormality (*n* = 17)	Abnormality (*n* = 9)	*p*-value
Donor artery			
DT	1.39 ± 0.96	1.43 ± 0.88	0.91
Speed	96.00 ± 61.40	132.56 ± 127.35	0.44
RT	2.60 ± 1.39	3.74 ± 3.55	0.38
Intensity	55.41 ± 37.80	41.33 ± 25.21	0.27
Receptor artery (proximal)			
DT	1.22 ± 1.10	1.68 ± 1.30	0.37
Speed	181.65 ± 137.18	129.33 ± 89.76	0.25
RT	3.12 ± 3.22	6.10 ± 2.61	0.02
Intensity	59.24 ± 19.79	54.89 ± 13.20	0.51
Receptor artery (distal)			
DT	1.35 ± 0.94	1.24 ± 0.83	0.77
Speed	226.06 ± 165.95	145.78 ± 124.94	0.18
RT	3.93 ± 3.66	4.26 ± 1.85	0.76
Intensity	55.06 ± 21.94	64.67 ± 17.14	0.23

**Figure 2:**
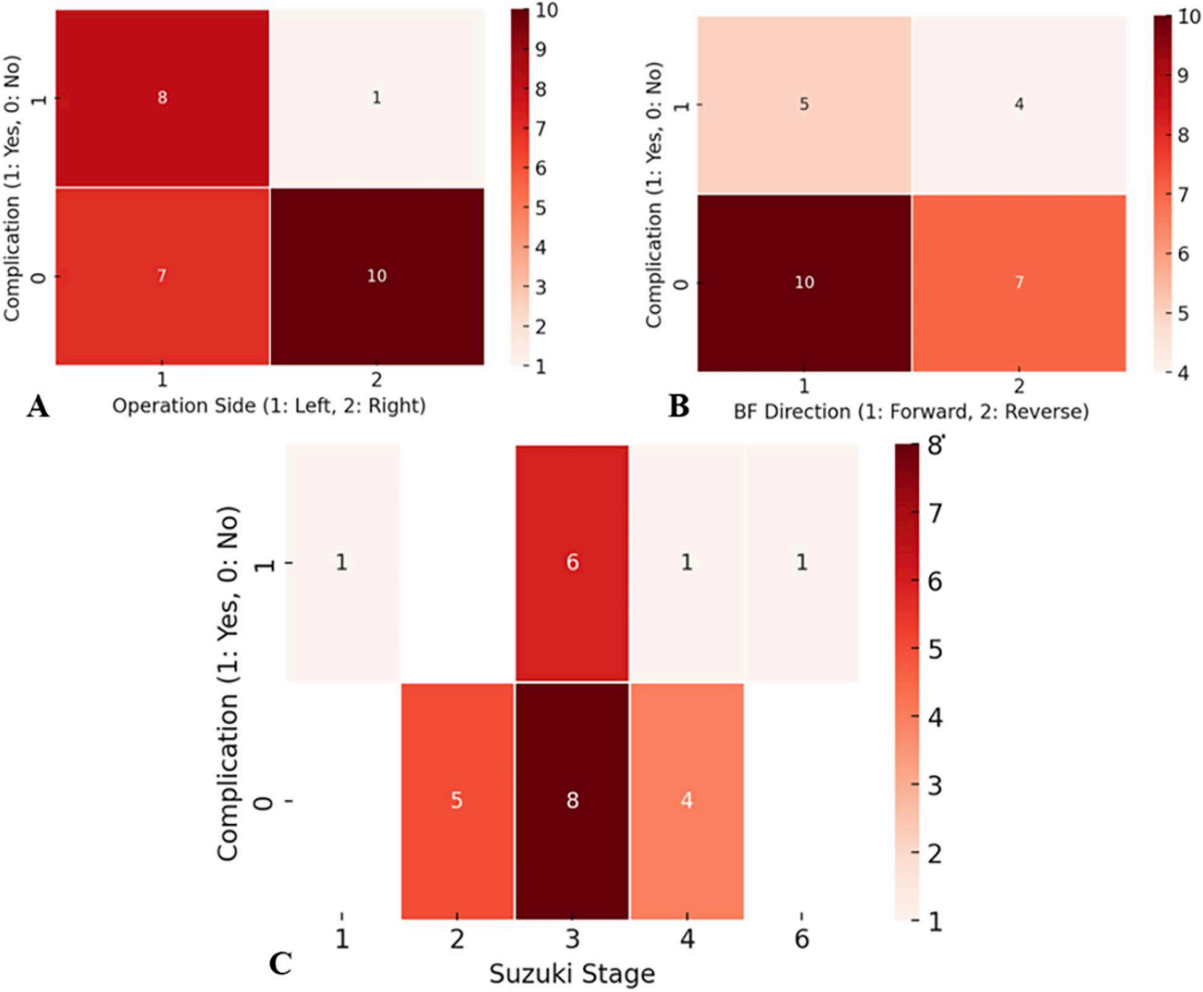
Factors associated with postoperative cerebral perfusion abnormalities. **(A)** Incidence of postoperative perfusion abnormalities according to bypass side. **(B)** Distribution of complications according to recipient artery flow direction. **(C)** Association between Suzuki stage and postoperative perfusion abnormalities.

Figure 2A illustrates that left-sided bypasses were associated with a significantly higher incidence of perfusion abnormalities compared to right-sided procedures (*p* = 0.02). Although the direction of recipient artery flow (anterograde vs. retrograde) was not statistically associated with perfusion abnormality rates (Figure 2B, *p* = 0.87), a clinical trend indicated a higher likelihood of radiographic ischemia in patients with retrograde flow.

Additionally, patients classified as Suzuki stage 3 exhibited a higher frequency of perfusion abnormalities; however, this trend did not reach statistical significance (*p* = 0.12), likely due to the limited sample size (Figure 2C).

### Predictive modeling of perfusion abnormalities

**Figure 3:**
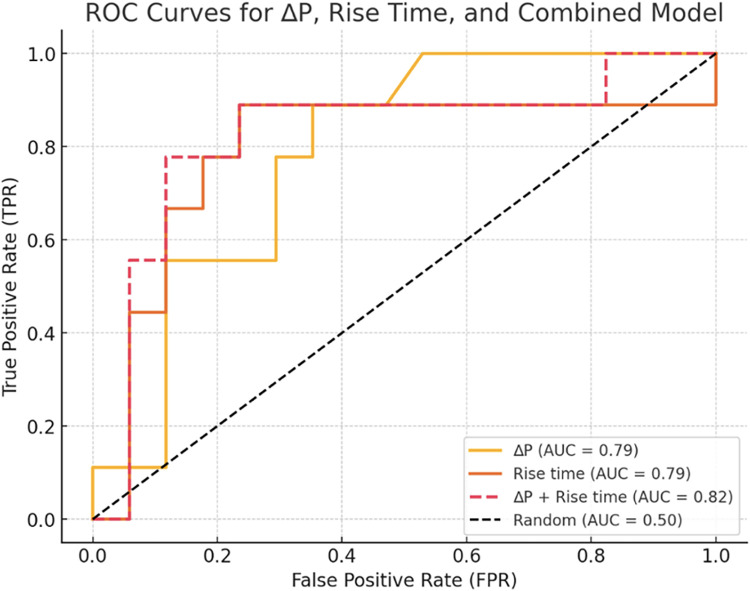
Receiver operating characteristic (ROC) analysis for prediction of postoperative perfusion abnormalities. ROC curves for ΔP, proximal recipient artery rise time (RT), and the combined model.

ROC curve analysis identified ΔP (>32 mmHg) and proximal RT as significant predictors of postoperative perfusion abnormalities (AUC = 0.79, 95% CI 0.59–0.94, for ΔP; AUC = 0.79, 95% CI 0.54–0.98, for proximal RT; Figure 3). For the proposed cutoff of ΔP > 32 mmHg, sensitivity was 77.8% and specificity was 64.7%. Combining these parameters improved predictive accuracy, achieving an AUC of 0.82 (95% CI 0.61–0.99), superior to either parameter individually. Given the limited sample size and number of outcome events, these predictive estimates should be interpreted cautiously.

## Discussion

Superficial temporal artery–middle cerebral artery (STA–MCA) bypass surgery remains a critical therapeutic option for restoring cerebral perfusion in Moyamoya disease (MMD). Nevertheless, postoperative cerebral perfusion-related complications, occurring in approximately 15%–35% of cases, continue to present substantial clinical challenges, impacting patient outcomes negatively (10, 11). Consequently, precise intraoperative prediction and early intervention for cerebral perfusion abnormalities have become essential focal points in cerebrovascular surgery (12). The current study evaluated the combined predictive utility of intraoperative fractional flow reserve (FFR) pressure wire and FLOW800 imaging to identify patients at elevated risk for postoperative perfusion disturbances.

Numerous modalities, including transcranial Doppler ultrasonography (TCD), laser speckle imaging (LSI), and near-infrared spectroscopy (NIRS), have previously been used for cerebral perfusion monitoring intraoperatively and postoperatively (13). While these techniques provide indirect insights into cerebral blood flow and tissue perfusion, they fail to directly quantify intravascular pressure gradients, thus limiting their predictive power for postoperative complications. In contrast, FFR pressure wire, a technology extensively utilized in coronary interventions, has recently been adapted for cerebrovascular surgeries, providing precise, real-time quantification of intravascular pressures and offering greater predictive accuracy for assessing cerebral hemodynamics (14, 15).

Similarly, FLOW800, a real-time optical imaging method utilizing indocyanine green (ICG) fluorescence, has gained prominence in cerebrovascular surgery due to its ability to visualize and quantify microcirculatory perfusion dynamically (16). Our results indicated that integrating FFR pressure wire with FLOW800 imaging significantly enhanced predictive accuracy for postoperative cerebral perfusion abnormalities, thereby facilitating more informed intraoperative decision-making.

At the same time, postoperative perfusion abnormalities after revascularization are unlikely to arise from a single factor. In clinical practice, they probably reflect an interaction among pre-existing vascular fragility, donor-recipient hemodynamic mismatch, recipient-vessel characteristics, temporary occlusion-related stress, and perioperative blood pressure management. We have therefore revised the present Discussion to place the pressure-wire and FLOW800 findings within this broader pathophysiological context rather than attributing postoperative events to one mechanism alone. This interpretation is also consistent with the observation that recipient artery pressure (P3) was lower in the complication group, suggesting that a large ΔP may partly identify territories that are already hemodynamically compromised before they are exposed to the new bypass inflow.

Our analysis revealed a significant association between increased pressure gradient across the bypass graft (ΔP) and the occurrence of postoperative perfusion abnormalities. A ΔP greater than 32 mmHg was associated with a higher risk of early postoperative perfusion-related events. However, ΔP should not be interpreted solely as a marker of hyperdynamic perfusion exceeding autoregulatory capacity. It may partly reflect differences in the underlying vascular condition of the recipient territory. Patients with poorer compensatory capacity, lower vascular compliance, and a more compromised distal vascular bed may be less able to accommodate newly introduced bypass flow, resulting in a larger pressure gradient after anastomosis. In this setting, a higher ΔP may identify a recipient circulation that is hemodynamically fragile and therefore more prone to postoperative perfusion-related abnormalities. At the same time, ΔP is unlikely to reflect a single mechanism alone and may also be influenced by lower recipient artery pressure, increased distal vascular resistance, and competitive flow between donor and native circulation. Therefore, ΔP should be interpreted as an integrated marker of hemodynamic imbalance rather than as a specific indicator of hyperperfusion alone. In other words, RT may be viewed as a practical surrogate of how efficiently the recipient microvascular bed accommodates newly introduced flow at the tissue level.

Additionally, prolonged rise time (RT) in the proximal recipient artery measured by FLOW800 imaging was significantly correlated with postoperative perfusion abnormalities. This finding likely reflects delayed microvascular filling and impaired local adaptation of the recipient territory after bypass. Among the FLOW800-derived parameters, RT may be particularly informative because it captures the speed of fluorescence inflow into the region of interest and may therefore be more sensitive to delayed microcirculatory adaptation than some of the other available parameters. Despite its utility, FLOW800 possesses inherent limitations, including sensitivity to intraoperative factors such as vessel depth, lighting conditions, ROI selection, and individual anatomical variability. Moreover, FLOW800 alone lacks intraluminal pressure information, underscoring its limitations as a standalone predictive tool. This may explain why pressure-based and FLOW800-based parameters, when interpreted together, are more clinically informative than either modality alone: one reflects the force delivered to the territory, whereas the other reflects how that territory receives and distributes that flow.

The combination of FFR and FLOW800 measurements offers complementary information, effectively bridging the limitations inherent to each modality individually. Pressure-wire assessment provides insight into hemodynamic driving forces, whereas FLOW800 reflects the tissue-level microcirculatory response. This dual-modality approach improved predictive accuracy in our cohort and supports the concept that postoperative perfusion abnormalities are better understood through combined assessment of driving pressure and tissue adaptation rather than either dimension alone.

Regarding the disease progression measured by the Suzuki stage, our study observed a trend wherein patients classified as stage 3 exhibited a higher incidence of postoperative perfusion abnormalities, although statistical significance was not attained due to limited sample size. One plausible explanation for this observation is that advanced stages of MMD may feature enhanced collateral circulation, providing some protection against abrupt postoperative hemodynamic fluctuations (17, 18). Conversely, significantly compromised cortical vasculature in advanced stages might limit responsiveness to bypass-induced perfusion changes (19).

Furthermore, the impact of recipient artery flow direction on postoperative outcomes warrants attention. Although no statistically significant association emerged between flow direction and postoperative perfusion abnormalities, we noted a pattern in which patients with anterograde recipient artery flow predominantly experienced transient clinical symptoms without radiographic ischemia, whereas retrograde flow was frequently linked to confirmed ischemic infarctions. This finding may suggest competitive flow dynamics in retrograde-flowing vessels, potentially restricting effective distal perfusion and raising ischemic risks postoperatively (20). Importantly, FFR pressure wire technology provides precise and objective quantification of pressure gradients along recipient vessels, offering a valuable tool for assessing these flow dynamics intraoperatively. Future studies with larger patient cohorts are necessary to confirm this relationship and refine preoperative and intraoperative management strategies accordingly.

### Clinical severity and endpoint selection

For this reason, the composite endpoint in the present study should be interpreted as an early perfusion-instability endpoint rather than as a single-pathway complication category.

Notably, although 9 patients developed postoperative perfusion-related neurological symptoms, only 3 demonstrated DWI-confirmed acute ischemic lesions, corresponding to a major complication rate of 11.5%. The remaining 6 patients had transient deficits that resolved within 2 weeks to 1 month. In this exploratory study, analyses were performed using the broader clinically relevant endpoint of perfusion abnormality (symptoms with or without imaging changes), because the number of major ischemic complications alone was insufficient for reliable modeling. We acknowledge, however, that transient neurological deterioration and DWI-confirmed ischemic lesions may represent partially different mechanisms and should be examined separately in larger cohorts.

### Exploratory nature and intraoperative management implications

At present, the most realistic application is therefore not immediate revision of the completed bypass, but intensified postoperative surveillance, stricter blood pressure control, and closer correlation with early neurological and imaging changes in patients identified as hemodynamically high risk.

This study represents an initial investigation of pressure dynamics in the moyamoya bypass system. Because the clinical impact of a large pressure gradient (ΔP) had not been established, no intraoperative corrective maneuvers were undertaken when high gradients were encountered. Therefore, under the current workflow, these measurements should primarily be interpreted as immediate intraoperative/postoperative risk stratification tools rather than as direct determinants of revision of the completed anastomosis. Based on the present findings, future prospective work may evaluate whether these measurements can help refine recipient selection, guide hemodynamic tailoring strategies, and individualize postoperative blood pressure management.

### Limitations and future directions

A prospectively designed study incorporating a contemporaneous comparison cohort, standardized perioperative hemodynamic targets, and longer-term functional follow-up would provide a more definitive test of whether these intraoperative markers can meaningfully alter clinical decision-making and outcome.

Several limitations should be acknowledged. First, this was a single-center retrospective exploratory study with a limited sample size, which may have reduced power to detect statistically significant differences in some subgroup analyses and increased the risk of overfitting in multivariable modeling. Because the present cohort included only 26 patients and 9 outcome events, robust multivariable analysis was considered unstable and was not pursued further. Second, no comparable non-monitored control cohort was available during the study period, and therefore the study could not directly evaluate whether the use of intraoperative FFR and FLOW800 monitoring itself altered postoperative outcomes. Third, although early postoperative perfusion abnormalities were captured, long-term functional and radiographic outcomes were not systematically analyzed. Future prospective multicenter studies with larger cohorts are needed to validate these findings and to clarify their long-term clinical significance.

In summary, our study provides initial evidence that integrating FFR pressure-wire assessment with FLOW800 imaging may improve early identification of patients at risk of postoperative cerebral perfusion abnormalities after STA–MCA bypass in moyamoya disease. Further research with larger cohorts is required to validate these findings.

## Conclusion

The combined use of fractional flow reserve (FFR) pressure-wire assessment and FLOW800 intraoperative imaging may improve identification of patients at risk of early postoperative cerebral perfusion abnormalities following STA–MCA bypass in moyamoya disease. A larger pressure gradient across the bypass graft and prolonged rise time (RT) in the proximal recipient artery appear to be promising candidate markers of hemodynamic imbalance. However, these findings should be interpreted as exploratory and require validation in larger prospective studies before routine translation into intraoperative decision-making.

## Data Availability

The original contributions presented in the study are included in the article/Supplementary Material, further inquiries can be directed to the corresponding author.
